# Critical care capacity and care bundles on medical wards in Malawi: a cross-sectional study

**DOI:** 10.1186/s12913-023-10014-8

**Published:** 2023-10-05

**Authors:** Emilia Connolly, Noel Kasomekera, Paul D. Sonenthal, Mulinda Nyirenda, Regan H. Marsh, Emily B. Wroe, Kirstin W. Scott, Alice Bukhman, Tadala Minyaliwa, Martha Katete, Grace Banda, Joia Mukherjee, Shada A. Rouhani

**Affiliations:** 1 Abwenzi Pa Za Umoyo/Partners In Health, PO Box 56, Neno, Malawi; 2 Division of Pediatrics, University of Cincinnati College of Medicine, 3230 Eden Ave, Cincinnati, OH 45267 USA; 3 Division of Hospital Medicine, Cincinnati Children’s Hospital, 3333 Burnet Ave, Cincinnati, OH 45229 USA; 4 Ministry of Health, P.O. Box 30377, Lilongwe 3, Malawi; 5 Partners In Health, 800 Boylston St Suite 300, Boston, MA 02199 USA; 6 Brigham & Women’s Hospital, Division of Pulmonary & Critical Care, 75 Francis St, Boston, MA 02115 USA; 7 Harvard Medical School, 25 Shattuck St, Boston, MA 02115 USA; 8 Adult Emergency and Trauma Centre, Queen Elizabeth Central Hospital, P.O. Box 95, Blantyre, Malawi; 9 University of Malawi College of Medicine, Private Bag 360, Chichiri, Blantyre 3, Malawi; 10 Brigham & Women’s Hospital, Department of Emergency Medicine, 75 Francis St, Boston, MA 02115 USA; 11 Brigham & Women’s Hospital, Division of Global Health Equity, 75 Francis St, Boston, MA 02115 USA; 12 Department of Emergency Medicine, University of Washington, Seattle, USA

**Keywords:** Critical care, Medical ward, Care capacity, Barriers

## Abstract

**Introduction:**

As low-income countries (LICs) shoulder a disproportionate share of the world’s burden of critical illnesses, they must continue to build critical care capacity outside conventional intensive care units (ICUs) to address mortality and morbidity, including on general medical wards. A lack of data on the ability to treat critical illness, especially in non-ICU settings in LICs, hinders efforts to improve outcomes.

**Methods:**

This was a secondary analysis of the cross-sectional Malawi Emergency and Critical Care (MECC) survey, administered from January to February 2020, to a random sample of nine public sector district hospitals and all four central hospitals in Malawi. This analysis describes inputs, systems, and barriers to care in district hospitals compared to central hospital medical wards, including if any medical wards fit the World Federation of Intensive and Critical Care Medicine (WFSICCM) definition of a level 1 ICU. We grouped items into essential care bundles for service readiness compared using Fisher’s exact test.

**Results:**

From the 13 hospitals, we analysed data from 39 medical ward staff members through staffing, infrastructure, equipment, and systems domains. No medical wards met the WFSICCM definition of level 1 ICU. The most common barriers in district hospital medical wards compared to central hospital wards were stock-outs (29%, Cl: 21% to 44% vs 6%, Cl: 0% to 13%) and personnel shortages (40%, Cl: 24% to 67% vs 29%, Cl: 16% to 52%) but central hospital wards reported a higher proportion of training barriers (68%, Cl: 52% to 73% vs 45%, Cl: 29% to 60%). No differences were statistically significant.

**Conclusion:**

Despite current gaps in resources to consistently care for critically ill patients in medical wards, this study shows that with modest inputs, the provision of simple life-saving critical care is within reach. Required inputs for care provision can be informed from this study.

**Supplementary Information:**

The online version contains supplementary material available at 10.1186/s12913-023-10014-8.

## Background

Low-income countries (LICs) account for a disproportionately high burden of critical illnesses such as trauma and sepsis, yet investments lack sufficient critical or intensive care capacity in these settings [1–5]. Longitudinal trends suggest that LICs have had slower decreases in the incidence of infection and sepsis, and complications from injury compared to high-income countries (HICs), and the lack of staff, resources, and infrastructure to respond to the burden of disease results in higher morbidity and mortality [3, 6, 7]. This has been highlighted with the COVID-19 pandemic, where delays in admission due to lack of resources were associated with double the mortality risk in low- and middle-income countries (LMICs) [8].

LICs struggle to replicate the model of intensive care units (ICUs) found in HICs due to the limited infrastructure, equipment, medications, and human resources required [3, 9–12]. Where ICU bed density in Europe ranges from 3.5 to 24.6 ICU beds/100,000 people [13], LIC ICU beds range from 0.1 to 0.4 with an estimated 0.1 in Malawi, a LIC in southeastern Africa [14–17]. However, the absence of ICUs does not mean a lack of critical illness. Instead, given the minimal ICU capacity in LICs like Malawi, critically ill patients are often treated in medical wards. Indeed, a district hospital in Malawi is more likely to treat a critically ill patient in the medical ward than in a dedicated intensive care unit, and 75% of central hospitals regularly treat critically ill patients on the wards [8]. These figures stress the need to ensure basic critical care provision in medical wards to address the significant burden of crucial illness in LICs.

Even when ICU capacity is sufficient, early recognition of critical illness and urgent interventions such as fluid resuscitation and antibiotics on medical wards can improve outcomes and avert the need for ICU admission in a decompensating patient [10, 17–29]. For inpatients in medical wards, modified triage or medical early warning systems based on physiological and physical parameters [18, 19, 22], oxygen delivery for childhood pneumonia [20], and modular critical care training programs for non-specialty staff [21, 30] have decreased mortality and morbidity.

Critical care in hospital wards must be measured, analysed with reporting, and improved to improve patient outcomes. Unfortunately, the lack of data on the capacity to treat critical illness—particularly outside of ICUs—in LICs hinders efforts to improve outcomes. A small number of assessment surveys have been conducted in LMICs, but most are limited to central hospitals in urban centres [10, 12, 16, 31–36]. Reliable data on resource availability and practice patterns, including critical care in medical wards, are essential to healthcare system planning and strengthening the capacity to care for critically ill patients [26, 34].

Malawi faces some of the highest known neonatal and maternal mortality rates as well as poor outcomes from critical illnesses such as sepsis due to a lack, in part, of resources and personnel [7, 37, 38]. Despite this burden, Malawi has only 4 ICUs in government hospitals, giving it a ratio of 0.1 ICU beds per million people [17]. These ICUs are concentrated in urban areas and include only 16 working ventilators [39, 40]. Thus, most critical care recognition and care are provided in medical wards in Malawi.

Using data collected during the Malawi Emergency and Critical Care (MECC) survey, we aim to describe the capacity to provide critical care in the medical wards of public sector secondary referral (district) and tertiary referral (central) hospitals in Malawi. We present the capacity of medical wards in domains of staff, systems, space, infrastructure, medications, and equipment and incorporate these domains into essential care bundles with an assessment of ICU status for medical wards per adapted from the World Federation of Intensive and Critical Care Medicine (WFSICCM) to identify opportunities for improvement to strengthen critical care throughout the health system.

## Methods

### Study design and setting

We characterised critical care capacity in medical wards in Malawian public sector referral hospitals by conducting a predefined secondary analysis of data from the MECC survey. The cross-sectional facility-based study was conducted in all four public sector central hospitals and a random sample of nine out of 24 Malawian public sector district hospitals in 2020. Full details of the MECC survey methodology, including sample size determination, are described elsewhere [40].

Malawi is a LIC with an estimated population of 19.6 million in 2022, over 80% of whom lives in rural areas [41]. Malawi’s healthcare system is organised into three tiers. The first tier is primary care which includes community and facility care at health centres and community hospitals providing basic medical care and staffed primarily by medical and nursing technicians (staff with two years of certificate training). The second tier is the district hospital, 29 in total in Malawi, staffed by non-residency trained physicians with internship training, clinical officers (advanced practice providers with three years of diploma training and one-year internship) and nurses (both diploma and degree level). District hospitals serve as the first referral level for primary levels with varying emergency and critical care infrastructure and services and provide non-specialized surgical care. The third tier is the central hospital. The four central hospitals in urban areas deliver more specialised emergency, medical, critical, and surgical care.

### Participants

At sampled facilities, the MECC enrolled clinical staff with at least one month of experience working in target units (e.g., emergency department outpatient department, general medical ward, high-dependency unit or ICU, or administration). Potential participants were identified and approached during site visits by the study team. All participants provided written informed consent. This analysis includes all participants from the MECC self-reporting working in general medical wards and hospital administration.

### Data collection

Data were collected using the MECC Survey instrument, which was designed to assess LIC facility service readiness to deliver emergency care and critical care (ECC) across three primary domains: (1) staff; (2) stuff – i.e., essential equipment, diagnostic tests, and medications; and (3) systems and space. Before formal administration, the instrument was piloted and evaluated for comprehensiveness, clarity, face validity, and reliability [38].

The instrument was administered through in-person interviews by study staff at sampled facilities between January 20th and February 18th, 2020. At each facility, data were obtained from one overall hospital administrator and three clinicians from each targeted unit, including the medical ward. Potential participants were identified through discussions with facility leadership, announcements during staff gatherings, and personal introductions while study staff visited different areas of the facility. To lessen the time burden on participants, questions on staff availability, ancillary support, available protocols, and quality improvement were only asked to one designated clinical lead at each unit. Data were collected and managed using REDCap electronic data capture tools [42].

The instrument contains several question structures. Signal function questions—items query the availability of a given resource or ability to perform an intervention—were rated on a scale of 1 to 3 (i.e., one is generally unavailable, 2 is some availability, and 3 is adequate availability). Questions on the frequency of activity were rated on a scale of 1 to 5 (i.e., one is almost never, two is infrequently, three is sometimes, four is frequently, and five is almost always).

Study staff prompted participants to identify all relevant barriers for any signal function questions generally rated unavailable or some availability. Barrier probe responses were coded into categories of infrastructure, absent equipment, broken equipment, stockout, personnel, training, user fees, and opening hours.

### Variables

We calculated estimates for medical wards by averaging the three participant responses within each ward. The mean score threshold of ≥ 2.5 was selected to ensure that a majority of respondents (2 out of 3) would have to agree for a signal function items to be considered “adequately available”. For yes/no questions, an item was considered present at a threshold of at least two participants with “yes” responses. For frequency questions, an article was considered present if the mean score among the three participants was at or above the threshold of 4 (out of 5) with the same rational as the signal function scores.

Medical ward barrier data are reported as a percentage calculated by taking the number of times each barrier category was identified divided by the total number of times participants generally responded unavailable or somewhat available to any signal function (i.e., number of times participants at the facility were asked to identify barriers).

#### Level 1 ICU definition

We used a composite measure to assess if medical wards met The World Federation of Intensive and Critical Care Medicine (WFSICCM) definition of a level 1 ICU [27]. Specifically, we applied WFSICCM criteria to define a level 1 ICU as any medical ward which meets the following nine criteria: (1) physicians with some experience in critical care available at least during the day, (2) higher nurse-to-patient ratios for critically ill patients, (3) at least twice daily reassessment of critically ill patients, (4) available pulse oximetry, (5) available cardiac monitoring, (6) available oxygen therapy, (7) available non-invasive respiratory support, (8) presence of basic quality improvement program, and (9) a transfer out policy.

#### Essential care bundles

We grouped items into essential care bundles by adapting lists of recommended inputs for essential care of critically ill patients as outlined in a consensus statement on essential emergency and critical care [27]. A complete list of signal function availability not captured in the essential care bundles or level 1 ICU criteria are reported in Supplemental Table 1.

### Missing data

For signal function and yes/no questions, we coded responses of “don’t know” as “generally unavailable” and “no”, respectively. This accounts for the fact that a service or resource is unlikely to be promptly provided for a critical patient when a clinician is unaware of its availability. Because the occurrence of an event is not contingent on clinician awareness, we took a different approach to frequency items—a response of “don’t know” was treated as missing data. For items with missing data from one participant (i.e., unit data were available for two participants,) we used the same thresholds for determining availability as above. If data were missing for two or more respondents in a unit, the item data for the team was considered missing/incomplete.

### Data analysis

Data analysis was conducted in Stata (Release 16). Medians and interquartile ranges summarised continuous and ordinal variables. Categorical variables were summarised using frequencies, proportions, and 95% confidence intervals. Barrier data were compared using Fisher’s exact test (two-tailed).

### Patient and public involvement

The research question and evidence generation were informed by Ministry of Health and hospital staff with need for assessment of critical care in medical wards and were involved in the design and conduct of the study. Hospital staff and administrators provided feedback on the pilot and clinical sensibility testing prior to completing data collection. Ministry of Health and the research team disseminated to hospital staff and administrators with further dissemination of the research in each facility. Patients were not involved as they were not directly part of the study [40].

### Ethical and checklist review

The MECC Survey protocol obtained ethical approval from the Partners Healthcare Institutional Review Board in Boston, USA (2019P003457) and the National Health Science Research Committee in Malawi (Protocol #19/05/2346, approval number 2346). The Malawi Ministry of Health also approved the study. The study was conducted by the Declaration of Helsinki guidelines and regulations [43]. We used the STROBE cross sectional checklist when writing our report [44].

## Results

### Hospital and respondent characteristics

A total of 13 hospitals participated in the MECC survey, including nine districts and four central hospitals. District hospitals had a median of 296 (IQR: 250 to 340) inpatient beds and 13,300 (IQR 10,000 to 21,000) annual inpatient admissions. The median inpatient beds and annual admissions for central hospitals were 911 (IQR: 487 to 1,239) and 35,100 (IQR 27,900 to 51,600), respectively.

We analysed the data from all 39 medical ward staff members who participated in the MECC survey (Table 1). At district hospitals, most respondents were nurses (70%) and clinical officers (22%), while at central hospitals, most respondents were nurses (67%), followed by doctors (25%). In both district and central hospitals, the respondents spent a median of 5 days a week working in the ward. When asked to identify areas in the hospital where critically ill patients are managed, six district and three central hospitals reported managing critically ill patients in the medical ward.

**Table 1:** Respondent and hospital characteristics

	**District hospitals**	**Central hospitals**
**Respondent characteristics**		
Total *n*	27	12
Nurse *n (%)*	19 (70%)	8 (67%)
Clinical officer *n (%)*	6 (22%)	1 (8%)
Doctor (with or without subspecialty training) *n (%)*	2 (7%)	3 (25%)
Number of days per week spent working on medical ward *median (IQR)*	5 (5 to 5)	5 (5 to 5.5)
**Hospital characteristics**		
Total *n*	9	4
Manage critically ill patients on medical wards *n (%)*	6 (67%)	3 (75%)

### Medical ward inputs

We examined medical ward inputs through the lens of the “4Ss” approach, where we grouped survey findings by i) Staff, ii) Systems, iii) Space, and iv) Stuff – i.e. supplies, infrastructure systems like oxygen delivery and equipment.

#### Staff

In all sampled district and central hospitals, medical providers and nurses were physically present in the wards 24 hours (h) a day (Table 2). There was 100% agreement between the scheduled numbers of nurses and actual staffing observed on the day of the survey. There was additional coverage available from providers on call inside the facility 24 h a day in 7 (78%) district hospitals and 1 (25%) central hospital. The median number of patients per nurse during the day of the visit was 10.1 (IQR 9.3 to 11.7) in district hospitals and 14.6 (IQR 13.4 to 17.5) in central hospitals. Few district hospitals (22%) had increased nurse-to-patient ratios for critically ill patients. The greatest availability of ancillary specialities in the district and central hospitals were clinical engineers, security, nutritionists, and physical therapists. Both district and central hospitals lacked respiratory therapists. Social workers and spiritual support were rarely available at district hospitals.

**Table 2:** Staff and systems to support critical care

	**District hospitals (*n* = 9)**	**Central hospitals (*n* = 4)**
**Staff availability on the medical ward**		
Clinicians are physically present in ward 24 hours (h) a day *n (%)*	9 (100%)	4 (100%)
Clinicians are on call inside facility 24 h a day *n (%)*	7 (78%)	1 (25%)
Nurses are present 24 h a day *n (%)*	9 (100%)	4 (100%)
Number of patients per nurse during day *median (IQR)*	10.1^a^ (9.3 to 11.7)	14.6^b^ (13.4 to 17.5)
Increased nurse to patient ratios for critically ill patients *n (%)*	2 (22%)	3 (75%)
Radiology results interpreted by radiologist *n (%)*	2 (22%)	4 (100%)
**Ancillary support services available to medical wards**		
Social workers *n (%)*	3 (33%)	2 (50%)
Security *n (%)*	6 (67%)	2 (50%)
Spiritual support *n (%)*	2 (22%)	2 (50%)
Nutritionists* n (%)*	8 (89%)	3 (75%)
Respiratory therapists* n (%)*	3 (33%)	1 (25%)
Physical therapists *n (%)*	8 (89%)	3 (75%)
Clinical engineers *n (%)*	8 (89%)	3 (75%)
**Systems availability on the medical ward**		
***Patient Observation***		
Wards with standard patient observation frequencies *n (%)*	8 (89%)	3 (75%)
Frequency of vital signs *n (%)*	9 (100%)	4 (100%)
Frequency of vital signs (hours) *median (IQR)*	24^a^ (12 to 24)	18 (10 to 24)
Frequency of vital signs for critically ill patients (hours) *median (IQR)*	12^a^ (2 to 12)	2 (2 to 2)
Wards with increased frequency of observations of critically ill patients *n (%)*	8 (89%)	3 (75%)
Formal system for identifying critically ill patients *n (%)*	0	1 (25%)
***Protocols available***		
Initial approach to ABCs (airway, breathing, circulation, etc.) and basic neurologic function *n (%)*	4 (44%)	2 (50%)
Medical resuscitation *n (%)*	4 (44%)	2 (50%)
Volume resuscitation* n (%)*	4 (44%)	1 (25%)
Sepsis management *n (%)*	3 (33%)	3 (75%)
Adjust volume resuscitation for malnourished or anemic patients *n (%)*	2 (22%)	1 (25%)
Asthma exacerbation management *n (%)*	3 (33%)	3 (75%)
Pneumonia management *n (%)*	3 (33%)	2 (50%)
Post-exposure prevention of STI/HIV, emergency contraception, counseling *n (%)*	9 (100%)	4 (100%)
Post exposure prophylaxis for health care workers *n (%)*	8 (89%)	3 (75%)
Hand-over when transferring patients from one care provider to another *n (%)*	5 (56%)	4 (100%)
Infection prevention and control *n (%)*	9 (100%)	4 (100%)
Managing hazardous exposures (including designated decontamination area) *n (%)*	8 (89%)	4 (100%)
Containment and disposal of sharps and biomedical waste *n (%)*	8 (89%)	4 (100%)
End of life care *n (%)*	1 (11%)	1 (25%)

^a^Data available from 8 hospitals

^b^Data available from 3 hospitals

#### Systems

Most medical wards reported seeing critically ill patients more frequently than other patients. Still, only one out of 13 hospitals had a standard protocol for the medical wards to identify critically ill patients (Table 2). For non-critical patients, vitals were checked a median of every 24 h (IQR 12 to 24) and 18 h IQR 10 to 24) at eight district hospitals with available data and central hospital medical wards, respectively. For critically ill patients, vitals were checked a median of every 12 h (IQR 2 to 12) and every 2 h (IQR 2 to 2) in the eight district hospitals and central hospital medical wards, respectively.

Most district and central hospital medical wards had protocols for post-exposure prevention for STI/HIV, emergency contraception, counselling, health care worker (HCW) post-exposure prophylaxis and infection prevention and control (Table 2). However, less than 50% of all medical wards had protocols for volume resuscitation, adjustment of volume resuscitation for malnourished or anaemic patients and end-of-life care. Less than 33% of district hospital wards had protocols for sepsis, asthma exacerbation, and pneumonia management.

#### Space

Fifty-six percent of district hospitals and 75% of central hospitals had a designated area for critically ill patients within the medical ward (Table 3). Only one of 9 district hospitals and two of 4 central hospitals had secure storage space in the ward for medications, including controlled substances. Only 75% or less of all wards had toilets accessible within the building for patients and staff.

**Table 3:** Availability of space, supplies, infrastructure systems, and equipment on general medical wards

	**District hospitals (*n* = 9)**	**Central hospitals (*n* = 4)**
**Space availability on the medical ward**		
Designated area for critically ill patients within medical ward *n (%)*	5 (56%)	3 (75%)
Toilets for patients and staff* n (%)*	5 (56%)	3 (75%)
Storage space (including for controlled substances) *n (%)*	1 (11%)	2 (50%)
**Critical supplies availability on the medical ward**		
Running water* n (%)*	4 (44%)	3 (75%)
Electricity* n (%)*	6 (67%)	4 (100%)
Paper chart *n (%)*	5 (56%)	3 (75%)
Electronic chart *n (%)*	0	0
Crash trolley or code cart with high-acuity equipment and supplies of various sizes *n (%)*	5 (56%)	1 (25%)
**Laboratory tests and diagnostics**		
Hemoglobin *n (%)*	9 (100%)	4 (100%)
Urine hcg *n (%)*	4 (44%)	4 (100%)
Glucose *n (%)*	9 (100%)	4 (100%)
Rapid HIV *n (%)*	8 (89%)	4 (100%)
Malaria rapid diagnostic test *n (%)*	6 (67%)	4 (100%)
Malaria smear *n (%)*	7 (78%)	4 (100%)
Full blood count* n (%)*	7 (78%)	4 (100%)
Coagulation profile (PT/PTT, INR)* n (%)*	0	0
Electrolytes* n (%)*	0	2 (50%)
BUN and creatinine* n (%)*	3 (33%)	2 (50%)
Lipase* n (%)*	0	1 (25%)
Cross matching for blood and blood products* n (%)*	9 (100%)	4 (100%)
Cardiac marker (e.g. troponin)* n (%)*	0	0
Blood cultures with sensitivities *n (%)*	1 (11%)	4 (100%)
Ultrasound for use in ward	2 (22%)	2 (50%)
**Oxygen delivery systems to the medical ward**		
Central piped system* n (%)*	0	0
Oxygen concentrator stored in the ward* n (%)*	5 (56%)	3 (75%)
Call for oxygen concentrator from central location if needed* n (%)*	5 (56%)	3 (75%)
Tanks that are stored in the ward* n (%)*	1 (11%)	2 (50%)
Call for tank from central location if needed* n (%)*	3 (33%)	3 (75%)
No adequately available oxygen delivery systems *n (%)*	4 (44%)	1 (25%)

#### Supplies, infrastructure systems and equipment

Running water was present in 44% of the district hospital and 75% of central hospital medical wards; electricity was present in 67% and 100%, respectively (Table 3). No medical wards had electronic charts. For access to a code cart with high-acuity equipment and supplies of various sizes on the medical wards, 56% of district and 25% of central hospital medical wards reported availability.

No district or central hospital medical ward had piped bedside oxygen outlets at the time of this assessment (Table 3). Most medical wards had available oxygen concentrators or oxygen tanks, but medical wards at 44% of district hospitals and 25% of central hospitals did not have reliable oxygen availability.

Regarding lab tests and diagnostics, medical wards at 87% of district hospitals and all central hospitals could test haemoglobin, glucose, rapid HIV, malaria smear, full blood count and cross-match for blood (Table 3). Only five total hospitals (3 districts (33%) and two central (50%)) could obtain electrolytes and BUN/creatinine for patients in medical wards. Blood cultures with sensitivities were available at only 1 district hospital medical ward but at all central hospital medical wards. No medical wards could test for coagulation profile or cardiac markers. Only four hospitals (2 districts (22%) and two central (50%)) had ultrasound machines available to use in the medical ward.

### Level 1 ICU criteria

None of the medical wards across the 13 hospitals met all WFSICCM criteria for a level 1 ICU (Table 4). District and central hospital medical wards met a median of 3 (IQR 1 to 3) and 6.5 (IQR 4 to 7.5) out of 9 possible criteria, respectively. Over 50% of district hospital wards had pulse oximetry, oxygen, and a basic quality improvement program, but none had cardiac monitoring, non-invasive ventilation, or physicians with some experience in critical care. No district and only three central hospital medical wards had clinicians who reassessed critically ill patients at least twice a day. Three of the four central hospital medical wards met at least six criteria, but only one had cardiac monitoring, and none had non-invasive ventilation.

**Table 4:** Level 1 ICU criteria and essential emergency and critical care components

	**District hospitals (*n* = 9)**	**Central hospitals (*n* = 4)**
**Level 1 ICU Criteria**^a^		
Physicians with some experience in critical care available at least during the day n (%)	0	3 (75%)
Higher nurse to patient ratios for critically ill patients n (%)	2 (22%)	3 (75%)
At least twice daily reassessment of critically ill patients *n (%)*	0	3 (75%)
Pulse oximetry (intermittent or continuous) *n (%)*	7 (78%)	4 (100%)
Cardiac monitoring *n (%)*	0	1 (25%)
Availability of oxygen *n (%)*	5 (56%)	3 (75%)
Availability of non-invasive ventilation *n (%)*	0	0
Basic quality improvement program^b^ *n (%)*	6 (67%)	4 (100%)
Policy and protocol for transferring patients to higher level of care *n (%)*	2 (22%)	2 (25%)
Number of criteria met *median (IQR)*	3 (1 to 3)	6.5 (4 to 7.5)
Wards meeting all criteria *n (%)*	0	0
**Essential Emergency and Critical Care Components**^c^		
***Identification of critical illness***		
Available method of blood pressure measurement *n (%)*	9 (100%)	4 (100%)
Pulse oximetry (continuous or intermittent) *n (%)*	7 (78%)	4 (100%)
Mental status exam *n (%)*	8 (89%)	3 (75%)
Standardized method (e.g. protocol/checklist) for identifying critically ill patients on the general medical ward *n (%)*	0	1 (25%)
Wards able to complete entire clinical bundle *n (%)*	0	1 (25%)
Number of signal functions in bundle adequately available *median (IQR)*	3 out of 4 (3 to 3)	3 out of 4 (2.5 to 3.5)
***Airway and breathing***		
Perform manual maneuvers to open the airway (e.g., jaw thrust, chin lift) *n (%)*	1 (25%)	0
Use of suction *n (%)*	8 (89%)	4 (100%)
Placement of oro- or nasopharyngeal airway device *n (%)*	0	1 (25%)
Availability of oxygen *n (%)*	5 (56%)	3 (75%)
Bag-valve mask ventilation *n (%)*	5 (56%)	3 (75%)
Wards able to complete entire bundle n (%)	0	0
Number of signal functions in bundle adequately available *median (IQR)*	3 out of 5 (2 to 3)	2.5 out of 5 (2 to 3)
***Circulation***		
External control of bleeding *n (%)*	9 (100%)	4 (100%)
Administration oral rehydration solution *n (%)*	9 (100%)	4 (100%)
Administration of IV fluids *n (%)*	9 (100%)	4 (100%)
Administration of adrenaline *n (%)*	7 (78%)	4 (100%)
Wards able to complete entire bundle n (%)	7 (78%)	4 (100%)
Number of signal functions in bundle adequately available *median (IQR)*	4 out of 4 (4 to 4)	4 out of 4 (4 to 4)
***Reduced level of consciousness***		
Protect unconscious patient from secondary injury *n (%)*	9 (100%)	3 (75%)
Diagnose and treat hypoglycemia *n (%)*	8 (89%)	4 (100%)
Administration of benzodiazepine for seizure *n (%)*	8 (89%)	4 (100%)
Management of extreme temperatures *n (%)*	9 (100%)	4 (100%)
Wards able to complete entire bundle n (%)	8 (89%)	3 (75%)
Number of signal functions in bundle adequately available *median (IQR)*	4 out of 4 (4 to 4)	4 out of 4 (3.5 to 4)
***Supportive care***		
Place peripheral IV *n (%)*	9 (100%)	4 (100%)
Place intraosseous line *n (%)*	0	0
Administration of IV or IM antibiotics *n (%)*	8 (89%)	4 (100%)
Administration IV opioid *n (%)*	5 (56%)	2 (50%)
Administer appropriate therapeutics for agitation *n (%)*	8 (89%)	3 (75%)
Administration of enteral nutrition *n (%)*	8 (89%)	4 (100%)
Reposition patient every four hours *n (%)*	5 (56%)	4 (100%)
Wards able to complete entire bundle *n (%)*	0	0
Number of signal functions in bundle adequately available *median (IQR)*	5 out of 7 (5 to 6)	5.5 out of 7 (4.5 to 6)

^a^Adapted from Marshall JC, Bosco L, Adhikari NK, Connolly B, Diaz JV, Dorman T, et al. What is an intensive care unit? A report of the task force of the World Federation of Societies of Intensive and Critical Care Medicine. Journal of critical care. 2017;37:270–6

^b^Defined as any quality improvement project related to the ward within the last 12 months

^c^Adapted from Schell CO, Khalid K, Wharton-Smith A, Oliwa J, Sawe HR, Roy N, et al. Essential Emergency and Critical Care: a consensus among global clinical experts. BMJ Glob Health. 2021;6(9)

### Essential emergency and critical care bundle components

Overall, hospitals met most of the inputs for essential emergency and critical care (Table 4). No medical ward could complete the entire essential care bundle for airway and breathing or supportive care. However, a median of 3 (IQR 2 to 3) of 5 components was available at district hospital medical wards for airway and breathing and 5 (IQR 5 to 6) of 7 components for supportive care. For identification of critical illness, no district hospital medical ward and only 1 (25%) central hospital medical ward could complete the entire care bundle. At least 75% of all medical wards could complete the entire care bundle for circulation and reduced level of consciousness.

### Reported barriers across essential critical care bundles

**Fig. 1:**
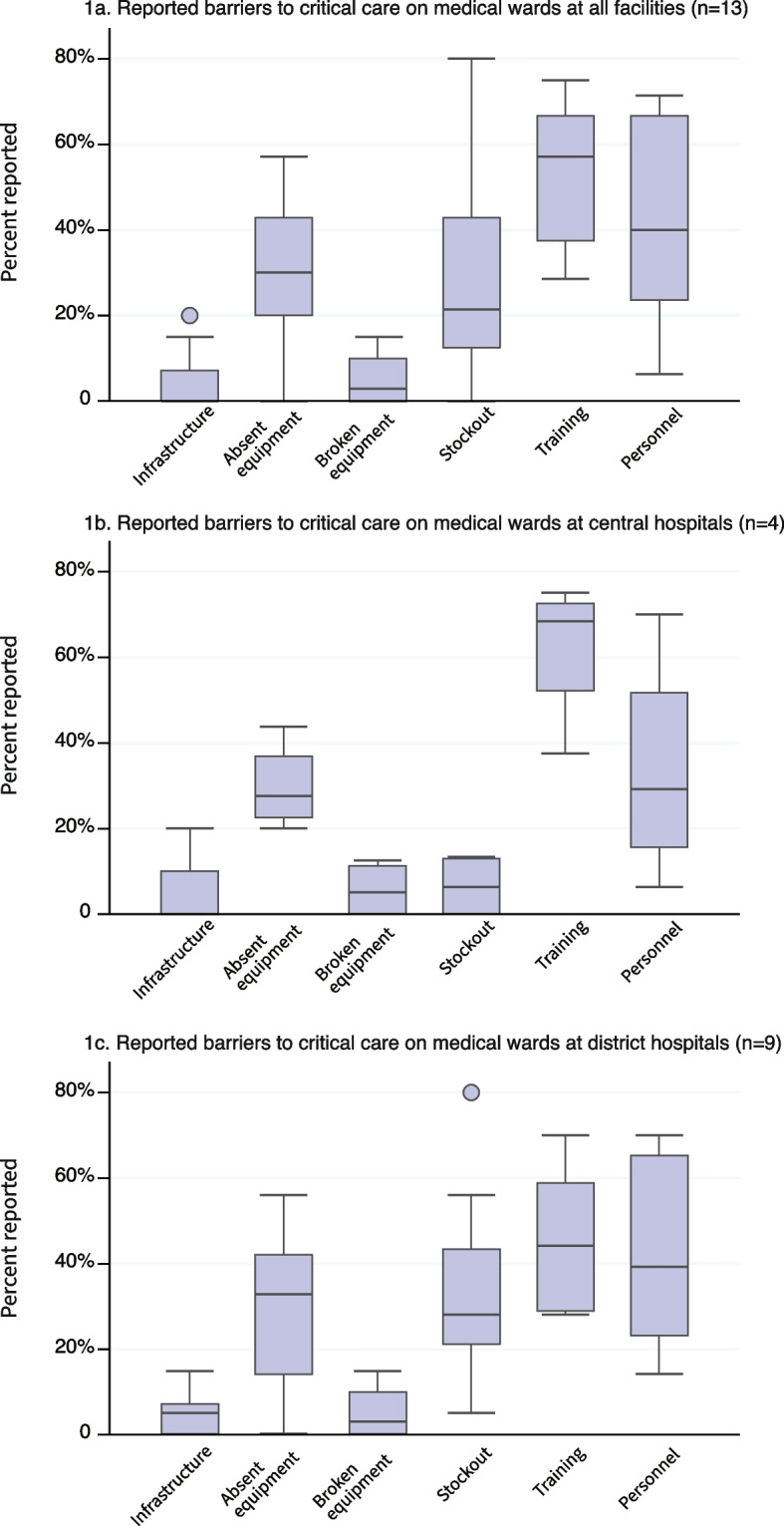
Box plots of percentage of times the six most common barrier categories were identified. The numerator was calculated as the number of times each category of barrier was identified by a participant at a unit for the signal functions included in the essential bundles*. The denominator was the number of times participants at a unit were asked to identify barriers (i.e. the number of times participants rated any of these signal functions as generally unavailable or somewhat available). *Mental status exam, perform manual maneuvers to open the airway manual maneuvers (e.g. jaw thrust, chin lift), use of suction, placement of oro- or nasopharyngeal airway device, availability of oxygen, bag-valve mask ventilation, external control of bleeding, administration oral rehydration solution, administration of IV fluids, administration of adrenaline, protect unconscious patient from secondary injury, diagnose and treat hypoglycemia, administration of benzodiazepine for seizure, management of extreme temperatures, place peripheral IV, place intraosseous line, administration of IV or IM antibiotics, administration IV opioid, administer appropriate therapeutics for agitation, administration of enteral nutrition, and reposition patient every four hours

Reported barriers across essential critical care bundles across all medical wards included absent equipment (30%, 95% CI: 20% to 43%), stockouts (21%, Cl: 13% to 43%), gaps in training (57%, Cl: 38% to 67%), and personnel (40%, Cl: 16% to 52%) (Fig. 1a). The least cited barriers were for broken equipment (3%, Cl: 0% to 10%) and infrastructure (0%, CI 0% to 7%). District hospital and central hospital medical wards had similar levels of reporting absent equipment (33%, Cl: 14% to 43% vs 28%, Cl: 23% to 37%) and broken equipment (3%, Cl: 0% to 10% vs 5%, Cl: 0% to 11%). District hospitals, compared to central hospital medical wards, had a higher proportion of stockouts (29%, Cl: 21% to 44% vs 6%, Cl: 0% to 13%) and personnel shortages (40%, Cl: 24% to 67% vs 29%, Cl: 16% to 52%) while central hospital medical wards reported a higher proportion of training barriers (68%, Cl: 52% to 73% vs 45%, Cl: 29% to 60%) (Figs. 1b and c). No differences in barriers between the district and central hospital medical wards were statistically significant.

## Discussion

To our knowledge, this is the first study to describe service readiness and capacity for critical care at the level of the medical ward in a LIC. Using signal function questions and composite assessments of ICU criteria and essential critical care bundles, we identified significant gaps across all four instrument domains—staff, stuff, systems, and space—highlighting the need for a holistic approach to strengthening essential critical care services in medical wards.

Our assessment of WFSICCM level 1 ICU criteria and essential critical care bundles provides valuable insight into the general critical care capacity in Malawian medical wards, highlighting relative strengths and areas where improvement is needed to deliver essential critical care. Even though none of the medical wards in the 13 central and district level hospitals surveyed met all criteria for the WFSICCM definition for level 1 ICU, many fundamental resources were available. Three of four medical wards in central hospitals met at least 6 of the 9 level 1 ICU criteria. District hospital wards met fewer level 1 ICU criteria with notable staff experience and availability gaps, quality improvement programs, and transfer-out policies. Most patients in Malawi are cared for at the district level [41, 45], suggesting an urgent need to provide the inputs to meet these gaps, with the identified low-cost inputs providing potential areas for care strengthening at the district level.

Examining signal functions as part of essential care bundles is vital to understanding if critical care can be delivered. For essential critical care bundles, no hospital could complete the bundles for airway, breathing, and supportive care, and only one central hospital could complete the bundle to identify a critical illness. These gaps limit the care that can be provided to patients as demonstrated in a recent study by Kayambankadzanja et al. [46] with up to 91% of patients not receiving essential critical care in Malawian hospitals. However, we found that observed gaps in signal functions with these bundles were relatively modest, including performing manual manoeuvres to open the airway, standardised methods for identifying critically ill patients on the medical ward, and intraosseous catheter placement. These areas offer a potential road map for improved service availability.

The gaps seen in the bundles reflect patterns observed across all signal functions. Compared to district hospitals, central hospitals' medical wards typically had more staffing and ancillary support, increased frequency of vital sign assessments, and more spaces and systems for the care of critically ill patients. About 60% of staffing posts are vacant in Malawi, most at the district level [45]. This results in high workloads for the available staff, which, coupled with a lack of resources, often delay or fail to identify and treat serious illness [3, 16, 28, 47]. We suggest that future implementation research and policies should examine ways to close the staffing gap, including ways to recruit and retain staff at district hospitals.

Our results are broadly consistent with two secondary analyses of facility-level data in emergency and critical care from the Malawi Service Provision Assessment (SPA) Survey 2013–2014 [31, 33]. Using the Emergency Triage Assessment and Treatment (ETAT) emergency-equipped definition, Johansson et al*.* [31] reported only four of 997 Malawi facilities with all 25 ETAT components; only one was a government hospital. However, of the 116 hospitals, 63.7% had between 20 and 24 ETAT components. Similarly, to this study, we found that while all ICU components are necessary to provide critical care to patients, many facilities were close to filling gaps of inputs across the domains. Kayambankadzanja et al. [33] found that government hospitals had a median resource availability of 48.4% (Cl: 40.6% to 64.1%) created by examining 63 essential emergency and critical care indicators. Moreover, in analysis of diagnostic availability from the SPA Survey, Yadav et al*.* [48] reported ~ 50% ultrasound and ~ 45% x-ray working availability in Malawian hospitals. Comparably to our findings, a lack of emergency guidelines (33.3%) was the most missing input, suggesting an area for future health system strengthening.

Observed barriers to stockouts at district hospital medical wards may be due to different supply chain systems utilised in the district and central hospitals. District and central hospitals procure most critical care medications through the public supply chain system but central hospitals can also procure through the private market from separate budgets [45, 49]. Although district and central hospital medical wards are designed to serve slightly different roles in the health system, our analysis focused on the basic essential inputs that should be available in all hospitals independent of more specialised care that may be needed on central referral hospital medical wards. From the observed barriers of stock out, we recommend further examination and strengthening of district and central hospital supply chains to ensure the availability of essential emergency and critical supplies at all health system levels.

Our results support an integrated approach to critical care strengthening across all instrument domains and underscore the importance of a holistic approach to assessing and improving critical care service availability. Many signal functions lacking availability are used across disease areas and care delivery settings. While the essential critical care bundles provide insight into specific areas of improvement to reach services readiness, the identified barriers in district and central hospital medical wards offer insight into how this could be done. Addressing these deficits can reinforce a severity-based approach to inpatient care agnostic to disease, patient, or speciality type [28]. This type of horizontal platform strengthens health systems and should be prioritised by funders and the government in the policy and intervention development [29].

### Limitations

This study provides important data on critical care in medical wards but has several limitations. As a single-country study, it is still being determined if the results are generalisable to other LICs, but likely similar gaps exist. Although the sample size was just 13 hospitals, this represents slightly under 50% of all public district and central hospitals. Similar findings in critical care assessments have been found in Tanzania [10], Kenya [50], India [32], Myanmar [51] and throughout sub-Saharan Africa and Asia [3, 34]. Although we examined the clinical cascade through clinician and nurse reporting, the actual care processes and outcomes were not observed, and our results could be influenced by reporting biases. Thirdly, though developed by consensus, the impact of the essential critical care bundles and the WFSICCM criteria have yet to be validated, with additional critical components that may be needed. Finally, this cross-sectional survey was completed before the Coronavirus (SARS-CoV-19) pandemic and there has likely been additions to district and central hospital ward signal functions and other inputs during this time that are not accounted for in this study.

## Conclusion

Despite current gaps in resources to consistently care for critically ill patients on medical wards, this study shows that with modest inputs within the domains of staff, medications and equipment, and systems and space, the provision of simple life-saving critical care is within reach for many central and district hospital medical wards. Findings from this study can help inform inputs required for care provision. Future process research could compare essential critical care in wards compared to high-dependency units and ICUs with health system readiness using cost-effectiveness or item response burden analyses. Outcome studies using essential care bundles for critical care to compare readiness estimates with specific barriers identified for action at a system level could identify and predict morbidity and mortality.

### Supplementary Information

**Additional file 1: Table S1.** Wards Reporting Adequate Ability to Perform Key Critical Care Signal Functions*

## Data Availability

The deidentified datasets used and/or analysed during the current study are available within Zenodo repository, 10.5281/zenodo.7950152 [52].

## Abbreviations

BUN: Blood urea nitrogen
CI: Confidence Interval
ECC: Emergency and Critical Care
ETAT: Emergency Triage Assessment and Treatment
HCG: Human chorionic gonadotropin
HCW: Healthcare worker
HIC: High-income country
HIV: Human Immunodeficiency Virus
H: Hours
ICU: Intensive care unit
IM: Intramuscular
INR: International normalised ratio
IQR: Interquartile range
IV: Intravenous
LIC: Low-income country
LMIC: Low- and middle-income country
MECC: Malawi Emergency and Critical Care
SPA: Service Provision Assessment
STI: Sexually Transmitted Infection
PT: Prothrombin time
PTT: Partial thromboplastin time
WFSICCM: World Federation of Intensive and Critical Care Medicine

## References

[1] Mock C, Kobusingye O, Joshipura M, Nguyen S, Arreola-Risa C (2005). Strengthening trauma and critical care globally. Curr Opin Crit Care.

[2] Cheng AC, West TE, Limmathurotsakul D, Peacock SJ (2008). Strategies to reduce mortality from bacterial sepsis in adults in developing countries. PLoS Med.

[3] Baelani I, Jochberger S, Laimer T, Otieno D, Kabutu J, Wilson I (2011). Availability of critical care resources to treat patients with severe sepsis or septic shock in Africa: a self-reported, continent-wide survey of anaesthesia providers. Crit Care (London, England).

[4] Vincent JL (2013). Critical care–where have we been and where are we going?. Crit Care (London, England).

[5] GBD 2019 Diseases and Injuries Collaborators (2020). Global burden of 369 diseases and injuries in 204 countries and territories, 1990–2019: a systematic analysis for the Global Burden of Disease Study 2019. Lancet (London, England)..

[6] James SL, Lucchesi LR, Bisignano C, Castle CD, Dingels ZV, Fox JT (2020). Morbidity and mortality from road injuries: results from the Global Burden of Disease Study 2017. Inj Prev.

[7] Rudd KE, Johnson SC, Agesa KM, Shackelford KA, Tsoi D, Kievlan DR (2020). Global, regional, and national sepsis incidence and mortality, 1990–2017: analysis for the Global Burden of Disease Study. Lancet (London, England).

[8] African COVID-19 Critical Care Outcomes Study (ACCCOS) Investigators (2021). Patient care and clinical outcomes for patients with COVID-19 infection admitted to African high-care or intensive care units (ACCCOS): a multicentre, prospective, observational cohort study. Lancet (London, England).

[9] Abdu M, Wilson A, Mhango C, Taki F, Coomarasamy A, Lissauer D (2018). Resource availability for the management of maternal sepsis in Malawi, other low-income countries, and lower-middle-income countries. Int J Gynaecol Obstet.

[10] Baker T, Lugazia E, Eriksen J, Mwafongo V, Irestedt L, Konrad D (2013). Emergency and critical care services in Tanzania: a survey of ten hospitals. BMC Health Serv Res.

[11] Henry JA, Frenkel E, Borgstein E, Mkandawire N, Goddia C (2015). Surgical and anaesthetic capacity of hospitals in Malawi: key insights. Health Pol Plan.

[12] Vukoja M, Riviello E, Gavrilovic S, Adhikari NK, Kashyap R, Bhagwanjee S (2014). A survey on critical care resources and practices in low- and middle-income countries. Global Heart.

[13] Adhikari NK, Fowler RA, Bhagwanjee S, Rubenfeld GD (2010). Critical care and the global burden of critical illness in adults. Lancet (London, England).

[14] Atumanya P, Sendagire C, Wabule A, Mukisa J, Ssemogerere L, Kwizera A (2020). Assessment of the current capacity of intensive care units in Uganda; a descriptive study. J Crit Care.

[15] Touray S, Sanyang B, Zandrow G, Dibba F, Fadera K, Kanteh E (2018). An assessment of critical care capacity in the Gambia. J Crit Care.

[16] Murthy S, Leligdowicz A, Adhikari NKJ (2015). Intensive care unit capacity in low-income countries: a systematic review. PLoS ONE.

[17] Morton B, Banda NP, Nsomba E, Ngoliwa C, Antoine S, Gondwe J (2020). Establishment of a high-dependency unit in Malawi. BMJ Glob Health.

[18] Kayambankadzanja RK, Schell CO, Nsanjama G, Mbingwani I, Kwazizira Mndolo S, Rylance J (2019). Inability to walk predicts death among adult patients in hospitals in Malawi. Emerg Med Int.

[19] Molyneux E, Ahmad S, Robertson A (2006). Improved triage and emergency care for children reduces inpatient mortality in a resource-constrained setting. Bull World Health Organ.

[20] Duke T, Wandi F, Jonathan M, Matai S, Kaupa M, Saavu M (2008). Improved oxygen systems for childhood pneumonia: a multihospital effectiveness study in Papua New Guinea. Lancet (London, England).

[21] Haniffa R, Lubell Y, Cooper BS, Mohanty S, Alam S, Karki A (2017). Impact of a structured ICU training programme in resource-limited settings in Asia. PLoS ONE.

[22] Kruisselbrink R, Kwizera A, Crowther M, Fox-Robichaud A, O'Shea T, Nakibuuka J (2016). Modified Early Warning Score (MEWS) identifies critical illness among ward patients in a resource restricted setting in Kampala, Uganda: a prospective observational study. PLoS ONE.

[23] Marshall JC, Bosco L, Adhikari NK, Connolly B, Diaz JV, Dorman T (2017). What is an intensive care unit? A report of the task force of the world federation of societies of intensive and critical care medicine. J Crit Care.

[24] Murthy S, Adhikari NK (2013). Global health care of the critically ill in low-resource settings. Ann Am Thorac Soc.

[25] O'Neill K, Takane M, Sheffel A, Abou-Zahr C, Boerma T (2013). Monitoring service delivery for universal health coverage: the service availability and readiness assessment. Bull World Health Organ.

[26] Riviello ED, Letchford S, Achieng L, Newton MW (2011). Critical care in resource-poor settings: lessons learned and future directions. Crit Care Med.

[27] Baker T, Schell CO, Petersen DB, Sawe H, Khalid K, Mndolo S (2020). Essential care of critical illness must not be forgotten in the COVID-19 pandemic. Lancet (London, England).

[28] Schell CO, Gerdin Wärnberg M, Hvarfner A, Höög A, Baker U, Castegren M (2018). The global need for essential emergency and critical care. Crit Care (London, England).

[29] Schell CO, Khalid K, Wharton-Smith A, Oliwa J, Sawe HR, Roy N (2021). Essential emergency and critical care: a consensus among global clinical experts. BMJ Glob Health.

[30] Opiyo N, English M. In‐service training for health professionals to improve care of seriously ill newborns and children in low‐income countries. Cochrane Database Syst Rev. 2015;5(5):1–15. 10.1002/14651858.CD007071.pub3 PMC4463987 25968066

[31] Johansson EW, Lindsjö C, Weiss DJ, Nsona H, Selling KE, Lufesi N (2020). Accessibility of basic paediatric emergency care in Malawi: analysis of a national facility census. BMC Public Health.

[32] Kashyap R, Vashistha K, Saini C, Dutt T, Raman D, Bansal V (2020). Critical care practice in India: Results of the intensive care unit need assessment survey (ININ2018). World J Crit Care Med.

[33] Kayambankadzanja RK, Likaka A, Mndolo SK, Chatsika GM, Umar E, Baker T (2020). Emergency and critical care services in Malawi: findings from a nationwide survey of health facilities. Malawi Med J.

[34] Leligdowicz A, Bhagwanjee S, Diaz JV, Xiong W, Marshall JC, Fowler RA (2017). Development of an intensive care unit resource assessment survey for the care of critically ill patients in resource-limited settings. J Crit Care.

[35] Nickerson JW, Adams O, Attaran A, Hatcher-Roberts J, Tugwell P (2015). Monitoring the ability to deliver care in low- and middle-income countries: a systematic review of health facility assessment tools. Health Pol Plan.

[36] Tripathi S, Kaur H, Kashyap R, Dong Y, Gajic O, Murthy S (2015). A survey on the resources and practices in pediatric critical care of resource-rich and resource-limited countries. J Intensive Care.

[37] Kawaza K, Kinshella MW, Hiwa T, Njirammadzi J, Banda M, Vidler M (2020). Assessing quality of newborn care at district facilities in Malawi. BMC Health Serv Res.

[38] Mgawadere F, Unkels R, Kazembe A, van den Broek N (2017). Factors associated with maternal mortality in Malawi: application of the three delays model. BMC Pregn Childb.

[39] Sonenthal PD, Masiye J, Kasomekera N, Marsh RH, Wroe EB, Scott KW (2020). COVID-19 preparedness in Malawi: a national facility-based critical care assessment. Lancet Glob Health.

[40] Sonenthal PD, Nyirenda M, Kasomekera N, Marsh RH, Wroe EB, Scott KW (2022). The Malawi emergency and critical care survey: a cross-sectional national facility assessment. eClinicalMedicine.

[41] Government of Malawi. Malawi Population and Housing Census Report - 2018. Zomba: National Statistical Office; 2019.

[42] Harris PA, Taylor R, Minor BL, Elliott V, Fernandez M, O'Neal L (2019). The REDCap consortium: building an international community of software platform partners. J Biomed Inform.

[43] World Medical A (2001). World medical association declaration of Helsinki. Ethical principles for medical research involving human subjects. Bullet World Health Org.

[44] von Elm E, Altman D, Egger M, Pocock S, Gotzsche P, Vandenbroucke J (2007). The Strengthening the Reporting of Observational Studies in Epidemiology (STROBE) statement: guidelines for reporting observational studies. Lancet.

[45] Government of the Republic of Malawi (2023). Health sector strategic plan III (2023–2030): Reforming for universal coverage.

[46] Kayambankadzanja RK, Schell CO, Mbingwani I, Mndolo SK, Castegren M, Baker T (2021). Unmet need of essential treatments for critical illness in Malawi. PLoS ONE.

[47] Gundo R, Mearns G, Dickinson A, Chirwa E (2019). Contextual issues that influence preparedness of nurses for critical care nursing practice in Malawi. Malawi Med J.

[48] Yadav H, Shah D, Sayed S, Horton S, Schroeder LF (2021). Availability of essential diagnostics in ten low-income and middle-income countries: results from national health facility surveys. Lancet Glob Health.

[49] Kaupa F, Naude MJ (2021). Barriers in the supply chain management of essential medicines in the public healthcare system in Malawi. Afr J Gov Dev.

[50] Cranmer JN, Dettinger J, Calkins K, Kibore M, Gachuno O, Walker D (2018). Beyond signal functions in global obstetric care: using a clinical cascade to measure emergency obstetric readiness. PLoS ONE.

[51] Seo DH, Kim H, Kim KH, Park J, Shin DW, Park JM (2019). Status of emergency signal functions in Myanmar hospitals: a cross-sectional survey. West J Emerg Med.

[52] Connolly E, Kasomekera N, Sonenthal PD, Nyirenda M, Marsh RH, Wroe EB (2023). Critical care in medical wards In Malawi: a cross-sectional study - data repository.

