# Supplementary material for: Critical care capacity and care bundles on medical wards in Malawi: a cross-sectional study
Source: BMC Health Serv Res. 2023 Oct 5;23:1062. doi: 10.1186/s12913-023-10014-8 (PMC10557270; doi:10.1186/s12913-023-10014-8)
Supplement: Supplementary file 1 — Additional file 1: Table S1. Wards Reporting Adequate Ability to Perform Key Critical Care Signal Functions* [file 12913_2023_10014_MOESM1_ESM.docx]

| **Table S1. Wards Reporting Adequate Ability to Perform Key Critical Care Signal Functions*** | | |
| --- | --- | --- |
|  | **District Hospitals**  **(n=9)** | **Central Hospitals**  **(n=4)** |
| ***Airway and Breathing*** |  |  |
| Placement of supraglottic device *n (%)* | 0 | 0 |
| Endotracheal intubation *n (%)* | 0 | 0 |
| Creation of surgical airway *n (%)* | 0 | 0 |
| Continuous pulse oximetry measurement *n (%)* | 0 | 0 |
| Administration of critical therapies for reactive airway disease *n (%)* | 8 | 2 |
| Perform needle decompression of tension pneumothorax *n (%)* | 0 | 0 |
| Placement of chest tube *n (%)* | 3 | 1 |
| Non-invasive ventilation *n (%)* | 0 | 0 |
| Invasive mechanical ventilation *n (%)* | 0 | 0 |
| ***Circulation*** |  |  |
| Adjust fluid resuscitation for malnutrition or severe anemia *n (%)* | 9 | 4 |
| Perform venous cutdown *n (%)* | 0 | 0 |
| Establish central venous access *n (%)* | 0 | 2 |
| Administer IV fluids *n (%) ** | 9 | 4 |
| Place urinary catheter *n (%)* | 9 | 4 |
| Perform packing and/or suture control *n (%)* | 8 | 3 |
| Apply arterial tourniquet *n (%)* | 1 | 2 |
| Apply pelvic binding or sheeting *n (%)* | 0 | 0 |
| Ability to perform safe transfusion (including protocols for appropriate ratios for massive transfusion) *n (%)* | 9 | 4 |
| Perform and interpret point of care ultrasound *n (%)* | 2 | 3 |
| ***Sepsis*** |  |  |
| Administration of IV or IM antibiotics *n (%)* | 8 | 4 |
| Administration of IV vasopressors *n (%)* | 0 | 1 |
| Perform diagnostic paracentesis *n (%)* | 7 | 4 |
| Bedside minor surgical techniques for source control (e.g., abscess, empyema) *n (%)* | 8 | 1 |
| ***Cardiovascular*** |  |  |
| Perform and interpret ECG *n (%)* | 0 | 3 |
| Administer aspirin for ischemia *n (%)* | 7 | 4 |
| Perform external defibrillation and/or cardioversion *n (%)* | 0 | 0 |
| Administration of adrenaline *n (%)* | 7 | 4 |
| Administer inotropes *n (%)* | 1 | 3 |
| Administer antiarrhythmics *n (%)* | 1 | 0 |
| Administer thrombolytics *n (%)* | 0 | 0 |
| Perform pericardiocentesis *n (%)* | 0 | 0 |
| ***Neurologic/Altered mental status*** |  |  |
| Check/administer glucose *n (%)* | 8 | 4 |
| Perform lumbar puncture *n (%)* | 9 | 4 |
| Administer locally appropriate antidote *n (%)* | 1 | 3 |
| Perform mental status exam *n (%)* | 8 | 3 |
| Administer appropriate therapeutics for agitation *n (%)* | 8 | 3 |
| Ability to provide physical restraints *n (%)* | 4 | 3 |
| Perform procedural sedation *n (%)* | 6 | 3 |
| Perform initial appropriate wound care *n (%)* | 9 | 3 |
| ***Supportive care and prevention of complications*** |  |  |
| Administer IV opioids *n (%)* | 5 | 2 |
| Administer IV sedatives (e.g., benzodiazepine, propofol) *n (%)* | 7 | 3 |
| Communicate with patient and/or families, including sharing poor prognoses *n (%)* | 8 | 4 |
| De-escalate care (e.g., stop treatments or remove life support) for patients with poor prognoses based on the expressed goals and wishes of the patient or their families n *(%)* | 0 | 0 |
| Administer DVT prophylaxis *n (%)* | 0 | 4 |
| Monitoring of nosocomial infections and antimicrobial resistance patterns *n (%)* | 0 | 2 |
| Frequently (at least every 4 hours) check electrolytes and adjust management based on results *n (%)* | 0 | 0 |
| Administer stress ulcer prophylaxis *n (%)* | 3 | 1 |
| **These functions are in addition to signal functions in Table 4* |  |  |
